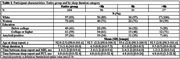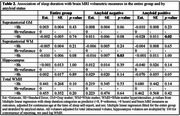# Self‐reported sleep duration in relation to brain MRI measures across pre‐symptomatic continuum in older adults age 90+

**DOI:** 10.1002/alz70857_104049

**Published:** 2025-12-25

**Authors:** Zarui A. Melikyan, Zeinah Al‐Darsani, Michael A. Yassa, Seyed Ahmad Sajjadi, Davis C. Woodworth, Claudia H. Kawas, María M. M. Corrada, Bryce A Mander

**Affiliations:** ^1^ University of California Irvine, Irvine, CA, USA; ^2^ University of California, Irvine, Irvine, CA, USA

## Abstract

**Background:**

Previous research found the association of long sleep with lower total brain and gray matter volumes in older adults. It is unclear, however, whether this association is modified by amyloid status in cognitively unimpaired individuals. We aim to examine the association of self‐reported sleep duration with brain MRI volumetric measures in cognitively unimpaired older adults age 90+ with and without significant amyloid positivity.

**Method:**

We included participants from ***The 90+ Study***, who were cognitively normal at the time of sleep report, had brain MRI which included a 3D T1w and a 2D FLAIR acquisition, and ^18^F‐florbetapir (amyloid) PET. Sleep duration was reported using Medical Outcomes Study Sleep Scale, and categorized as <8, 8=reference, >8 hours. We used sleep report closest to MRI. We analyzed continuous MRI volumetric measures of supratentorial gray (SGM) and white (SWM) matter, hippocampus, and white matter hyperintensities. We fitted multiple linear regression for the entire group and stratified by amyloid PET status. Models with sleep duration as predictor and brain MRI measures as outcomes were adjusted for age and sex.

**Result:**

In 104 participants mean age at sleep report was 93 years, average time between sleep report and MRI was 2 months, 36% were amyloid positive (Table 1). In the amyloid negative subgroup, those with >8 hours of sleep, compared to 8 hours, had lower SWM volume (Estimate=‐0.016, *p* = 0.03) (Table 2). In the amyloid positive subgroup, those with >8 hours of sleep, compared to 8 hours, had lower SGM volume (Estimate=‐0.028, *p* = 0.02).

**Conclusion:**

In this exploratory analysis long sleep is related to lower SWM volume in amyloid negative, and to lower SGM volume in amyloid positive older adults with normal cognition. In the amyloid negative group long sleep, which could indicate poor sleep quality, may be related to vascular pathologies, which in turn, affect white matter integrity. In amyloid positive group lower gray matter volume might be more related to the amyloid accumulation.